# Inverse design of structural color: finding multiple solutions *via* conditional generative adversarial networks

**DOI:** 10.1515/nanoph-2022-0095

**Published:** 2022-05-16

**Authors:** Peng Dai, Kai Sun, Xingzhao Yan, Otto L. Muskens, C. H. (Kees) de Groot, Xupeng Zhu, Yueqiang Hu, Huigao Duan, Ruomeng Huang

**Affiliations:** Faculty of Engineering and Physical Sciences, University of Southampton, SO17 1BJ, Southampton, UK; School of Physics Science and Technology, Lingnan Normal University, 5240481, Zhanjiang, China; National Engineering Research Center for High Efficiency Grinding, College of Mechanical and Vehicle Engineering, Hunan University, 410082, Changsha, China

**Keywords:** deep learning, Fabry–Pérot cavity, generative adversarial networks, inverse design, one-to-many problem, structural color

## Abstract

The “one-to-many” problem is a typical challenge that faced by many machine learning aided inverse nanophotonics designs where one target optical response can be achieved by many solutions (designs). Although novel training approaches, such as tandem network, and network architecture, such as the mixture density model, have been proposed, the critical problem of solution degeneracy still exists where some possible solutions or solution spaces are discarded or unreachable during the network training process. Here, we report a solution to the “one-to-many” problem by employing a conditional generative adversarial network (cGAN) that enables generating sets of multiple solution groups to a design problem. Using the inverse design of a transmissive Fabry–Pérot-cavity-based color filter as an example, our model demonstrates the capability of generating an average number of 3.58 solution groups for each color. These multiple solutions allow the selection of the best design for each color which results in a record high accuracy with an average index color difference Δ*E* of 0.44. The capability of identifying multiple solution groups can benefit the design manufacturing to allow more viable designs for fabrication. The capability of our cGAN is verified experimentally by inversely designing the RGB color filters. We envisage this cGAN-based design methodology can be applied to other nanophotonic structures or physical science domains where the identification of multi-solution across a vast parameter space is required.

## 1 Introduction

Retrieval of structural arrangements for a desired optical performance has been the fundamental challenge in nanophotonics inverse design [1]. This challenging task requires searching a range of geometrical parameters in an ample design space. For the past decades, sophisticated methods have been developed to arrive at optimized solutions using heuristics-based approaches such as particle swarm [2, 3], genetic algorithms [4], [5], [6], and gradient-based approaches [7, 8]. Often, the intuition and experience of the designer have been critical in obtaining rational operational principles with the advantage that the performance of such structures can be understood [7, 9], [10], [11], [12]. However, such a rational approach can be rather unsatisfactory as human intuition will not always guarantee an optimal design with the highest possible performance.

The recent remarkable development of deep learning (DL) has offered great promise in solving some of the critical challenges faced in nanophotonics [1, 13], [14], [15]. DL is a subset of machine learning technology inspired by animal brains’ biological neural networks [16]. Through a training process that involves iterative optimization of network parameters *via* gradient descent [17], the DL model, which contains a few artificial neuron layers, can learn the non-linear relations between the photonic structure and optical response. In recent years, it has found many successful applications in photonic research such as chiral metamaterials [18, 19], wavefront engineering [20], [21], [22], and structural color [23], [24], [25], [26], [27]. An exhaustive overview of this field of study can be found in the review literature [14, 15].

In the inverse design of nanophotonic structures, the DL regression models have been utilized to predict the potential solution of optical response and achieved success to some extent under some specific regions and constraints [9, 25, 28], [29], [30], [31]. One of the main difficulties for inverse design is the “one-to-many” mapping characteristic where one target optical response can be produced by multiple structures. A direct inversion of the artificial neural network (ANN) to predict the design usually fails as the training will not converge due to the existence of several possible solutions [32]. To circumvent the challenges related to the “one-to-many” mapping, tandem networks architecture is proposed [9]. This type of network consists of two sub-networks: an actual inverse network that takes the optical response and outputs the desired design and a pre-trained forward network that evaluates the outputs and generates optical responses. The tandem architecture allows direct comparison in the physics domain and avoids confusion from different designs that lead to a similar optical response. In previous work, we adopted such an architecture for the inverse design of a Fabry–Pérot-cavity-based structural color with high design accuracy [33]. Despite the success in tackling the convergence problem using this tandem network, the one-to-many problem leaves us with some critical issues. A prominent limitation of the tandem approach resides in the existence of *inaccessible solutions* [15] or a *dead zone* [34], which means that some possible solutions or solution spaces are discarded or unreachable during the network training process. In consequence, only a singular solution can be identified for each target optical response. The collapse of the network onto a set of singular solutions significantly limits the solution diversity and could even prevent reaching an optimal solution as the selected solution is not necessarily the best performing one. Therefore, developing an approach that enables identifying multiple solutions in different local/global optima can be extremely beneficial in achieving higher inverse design accuracy/performance. In addition, it will also provide more freedom in the actual fabrication process to select designs that fits the manufacturing requirement or facility limitation. For instance, a solution with the smaller dimensions could be selected to realize denser device integration while a large-dimension solution could be more suitable when the available facilities have certain resolution limitations. Furthermore, the performance decay induced by the fabrication error can also be mitigated *via* manufacturing a device with a less parameter-sensitive solution.

Compared with other works that employed the generative models (e.g., generative adversarial networks (GAN) and variational autoencoder (VAE)) to design the 2D metasurface [35], [36], [37], [38], [39], we introduced the GAN model [40] into the parameter-based nanophotonic inverse design, which provides an approach fully addressing the one-to-many problem. GAN is one of the most popular generative models that have been widely used in computer vision and image synthesis [41]. It consists of two networks, a generator and a discriminator, which contest with each other during the training process to create authentic images [42]. Due to its unique ability to reveal the hidden distribution behind enormous training datasets, GAN has been used by optical scientists to inversely design metasurfaces and diffractive optical devices with a high degree of freedom [22, 43], [44], [45], [46]. Here, we investigate the potential of applying GAN in identifying multiple solutions for one target optical response. We argue that the geometrical distribution in the training dataset is a key hidden parameter that can be utilized in GAN to avoid *dead zone* and identify multiple solutions.

We conduct an inverse design of a transmissive Fabry–Pérot (F–P) cavity-based structural color filter device using GAN as a demonstration. The structural color filter can display colors by selectively transmitting or reflecting the light with a specific wavelength by varying geometry. It has received enormous interest from research communities owing to its potential applications in display, full-color printing, optical encryption and solar cells [47], [48], [49]. The reasons for using such a system are threefolds: Firstly, the inverse design of the tri-layer F–P cavity structure (Ag–SiO_2_–Ag in this work) is a typical one-to-many problem as the transmissive peaks would appear periodically at the same wavelength as the increase of resonant cavity length [50, 51]; Secondly, as a relatively simple structure with the well-established analytical model [52], the performance of the developed GAN can be effectively investigated and evaluated. Thirdly, the design quality can be precisely quantified by the color difference index Δ*E*_2000_ [53] (denoted as Δ*E* below), which is calculated from two CIELAB vectors and has a physical meaning in statistics compared to mean squared error (MSE). After training, our GAN produces an average of 3.58 solution groups for each color in the testing set, which covers 93.9% of all ground truths. These multiple solutions allow the selection of the best design for each color which results in the lowest average color difference Δ*E* of 0.44 that has ever been reported for the inverse design of structural color. The identification of multiple solution groups is verified experimentally by fabricating different multilayer structures to demonstrate the same color.

## 2 Results and discussion

### 2.1 Problems of tandem network

**Figure 1:**
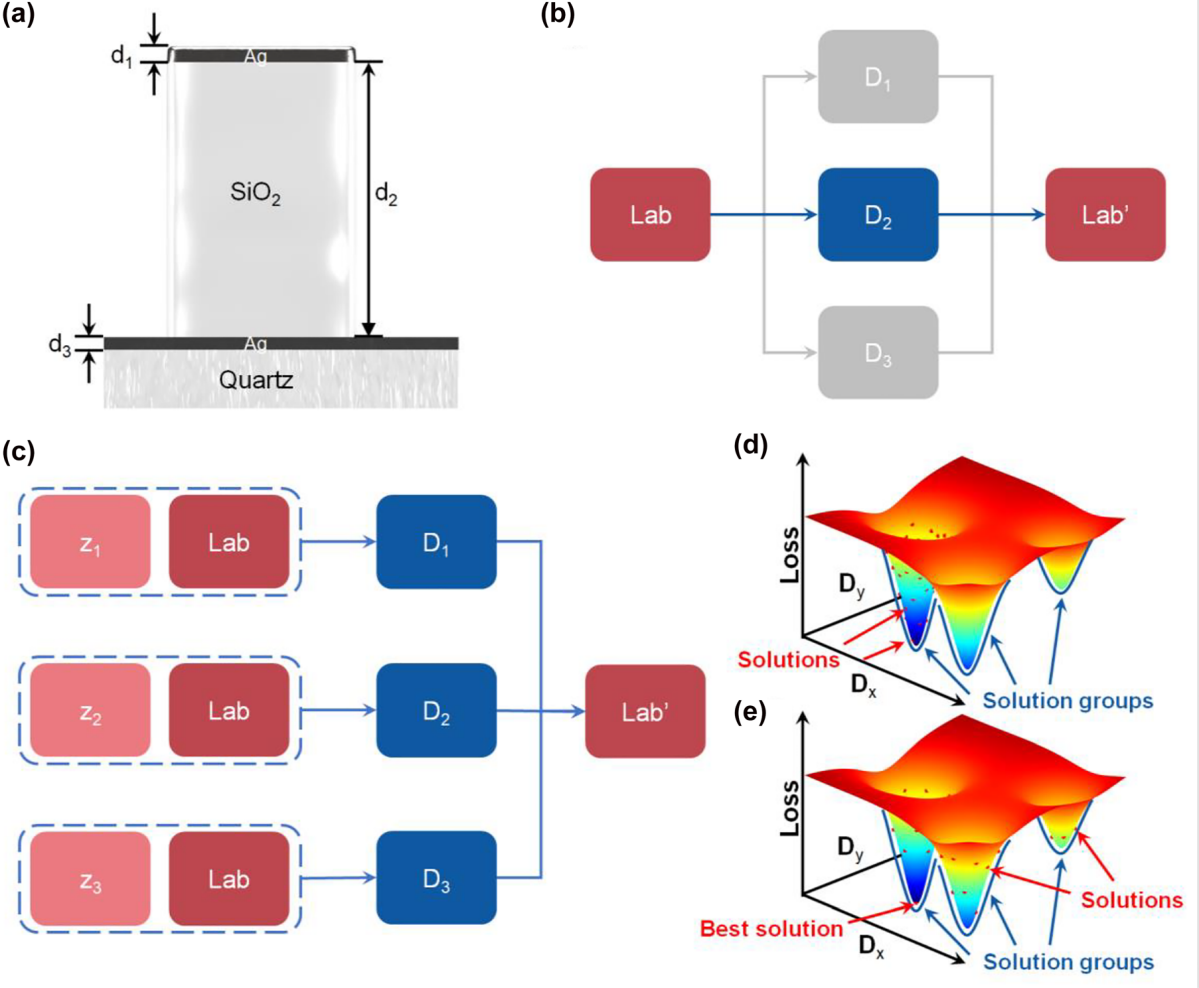
The schematics elaborate on the approach to tackling the one-to-many problem. (a) The schematic of the transmissive color filter structure in this demonstration. (b) The convergence schematic of the tandem network. (c) The schematic of converting the one-to-many problem to three one-to-one problems. (d and e) The cartoon loss landscape schematics of loss versus **D** when the output is degenerated (d), and the problem is well addressed (e).

We start by elaborating on the critical *dead zone* problem that the tandem network faces when performing inverse design. Taking the inverse design of a transmissive F–P-cavity-based structural color device as an example, the structure (shown in Figure 1a) has two reflective metal layers (Ag), which are separated by a lossless dielectric (SiO_2_) and stacks on a quartz substrate. The function of color filter is achieved by multiple round-trip phase delays of electromagnetic waves in the F–P resonator. By tuning the thickness of each layer (d_1_, d_2_, and d_3_, denoted as a single design vector **D** below), the resonant peak can be controlled to produce different colors. In a typical one-to-many problem, one color, represented by the CIELAB vector **Lab**, could be produced by several different structures **D**_1_, **D**_2_, and **D**_3_. When this color (**Lab**) is fed to a tandem network, only one of these solutions will be selected. The other solutions will be discarded to minimize the loss during the training, as illustrated in Figure 1b. This solution degeneracy is due to the inherent contradiction between the one-to-one mapping nature of ANNs and the one-to-many mapping problem.

One potential approach to resolve this contradiction is to introduce a latent vector **z**. The value of this latent vector can be sampled from a specific distribution (e.g., uniform or normal distribution) and assigned to the **Lab** (as shown in Figure 1c). Therefore, the one color can be split into three inputs by the variation of this latent vector and generates three one-to-one mappings that the tandem network can handle. However, these three inputs could still degenerate into very similar solutions within one solution group. The cartoon schematic of such degeneracy is illustrated in Figure 1d where the loss versus **D** landscape is plotted. The three sunken areas represent the three potential solution groups (outlined by blue lines) for this color. Different inputs can still be mapped into one sunken area, resulting in the degeneracy of the solution groups (depicted as the red dots). This behavior will be demonstrated and discussed later in the paper. Therefore, more constraint is required to force the network to search in the entire **D** space so all three solution groups can be identified (shown in Figure 1e). This additional constraint can be realized by generating a dataset distributed across the entire **D** space and forcing the network to learn such distribution. This means that alongside the effort to minimize the color difference, the distribution distance between the true thickness and the generator’s predicted thickness should also be minimized during the training process.

### 2.2 cGAN network design and training

**Figure 2:**
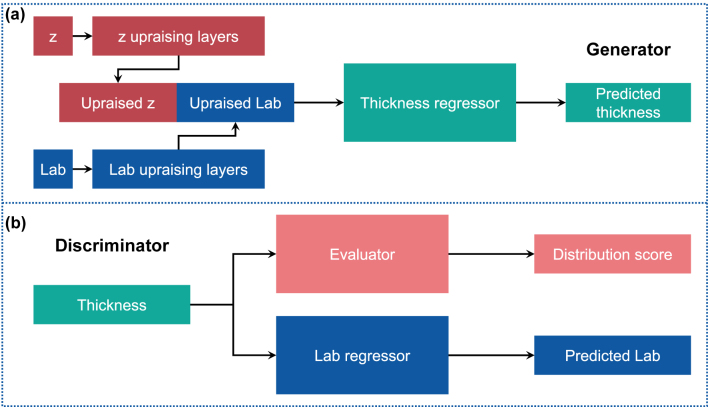
The architectures of the cGAN are used in the work. (a) Generator. (b) Discriminator.

The conditional generative adversarial networks [54] (cGAN) is a perfect tool for this task. cGAN usually has two parts, a discriminator and a generator. The generator takes a latent vector and the design target (condition) as inputs and outputs the generated data (also known as fake data). The discriminator evaluates the performance and distribution of the fake data and produces losses for backpropagation to shape the fake data close to the real data (training data). Figure 2 illustrates the network architectures used in this paper (details are shown in Figure S1). The generator comprises three sub-networks: Lab upraising layers, *z* upraising layers, and thickness regressor. The upraising layers are responsible for raising the dimensions of **Lab** and **z** from 3 and 2 to 128. The two upraised vectors are concatenated and put into the thickness regressor, which has 256 neurons per hidden layer and a sigmoid function at the end of the output layer, to generate the predicted thicknesses. The discriminator has two parallel networks: evaluator and Lab regressor. The evaluator evaluates the thickness data from the dataset (real) and generator (fake) and outputs two distribution scores. The difference between real and fake scores measures the distribution difference between ground truths and predicted data. The pre-trained Lab regressor takes thickness as input and returns the corresponding color (**Lab**).

**Figure 3:**
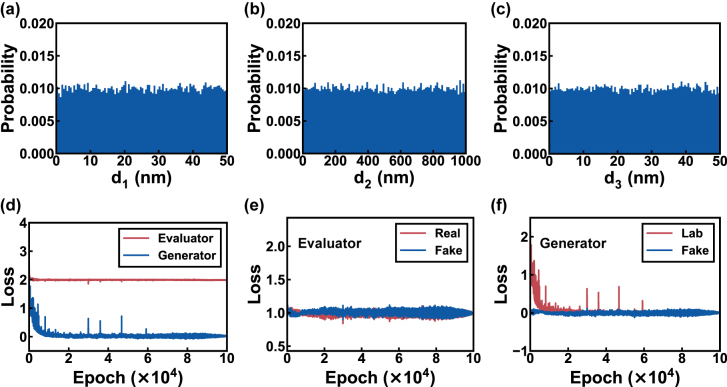
The dataset distribution and cGAN training loss curves. (a–c) The thickness histograms of the entire dataset. (d) The training loss curves of the generator (blue) and evaluator (red). (e) The curves of evaluator loss components including the real (red) and fake (blue) scores. (f) The MSE between **Lab** predicted by generator and ground truth (red), and generator’s fake score (blue).

As mentioned above, during the training of cGAN, a reference data (real data) distribution is required so the generator could learn the distribution from these data *via* the evaluator. The dataset was generated by randomly sampling 50,000 different combinations of layer thicknesses (**D**) using a uniform distribution. We set the sampling range of 0–50 nm for the Ag films and 0–1000 nm for the SiO_2_ film. As a thicker Ag layer will significantly reduce the color brightness while too thick SiO_2_ can cause many resonances in the visible spectrum and induce color degeneration and crosstalk, we believe that the sampling ranges and distribution are sufficient to cover the requested **D** space for diverse solution groups. The thickness distributions are shown in Figure 3a–c, in which the thicknesses are uniformly divided into 100 groups, and the bars indicate the probability density of different groups. It is worth mentioning that other thickness distributions may also be applied as the reference distribution, but the uniform one is more straightforward for us due to its accessibility in implementation and excellent color coverage. These structural designs were then turned into transmission spectra *via* the transfer matrix method and converted into HSV (hue, saturation, and value) and CIELAB color models. All generated colors are plotted in the CIE 1931-xy chromaticity diagrams in Figure S2. The discussion of relationships between geometry parameters and the color is provided in Section 2 of Supplementary Materials. The obtained dataset is divided into three groups for training (40,000), validation (5000), and testing (5000).

Before training our cGAN model, we firstly trained a Lab regressor to achieve forward modeling of the color **Lab** from a structure **D**. The detailed training process of this Lab regressor is provided in Supplementary Materials (Table S1 and Figure S4). The trained Lab regressor demonstrates an average color difference (Δ*E*) of 0.19 from the testing set. The significance of Δ*E* can be classified into five groups: (1) Δ*E* < 1, no discernable color difference; (2) 1 < Δ*E* < 2, the difference can be observed by experienced persons; (3) 2 < Δ*E* < 3.5, the difference can be observed by inexperienced persons; (4) 3.5 < Δ*E* < 5, clear difference can be noticed; (5) 5 < Δ*E*, two different colors are observed [55]. In the training of cGAN, both the color and thickness distribution differences are used in the loss function to apply backpropagation. We used Wasserstein distance as the distribution loss and applied spectral normalization to both evaluator and generator to improve the training stability [56]. The updating in the training process was alternatively performed on the generator and evaluator to minimize the hinge adversarial loss [57], [58], [59]. Details of the training process and the definitions of loss functions are provided in the Supplementary Materials (Table S2 and Equation S1–S3).

The training loss curves of the generator and evaluator are shown in Figure 3d. The loss of generator demonstrates a clear drop to close to zero, indicating the significant improvement of its predicting capability during the training process. The loss of evaluator compares the fake and real score and remains steadily at *ca.* 2 during training. To better understand this behavior, we plot the value of real and fake scores as a function of the training epoch in Figure 3e. It can be observed that the real and fake scores are close, indicating a balanced performance improvement for the generator and evaluator. This property is essential in cGAN training to ensure a balanced development of the generator and evaluator, which will be discussed in detail in the following section. Figure 3f displays the evolution curve of the generator. The Lab loss (red line), which is the MSE between prediction and ground truth of **Lab**, dropped fast in the first ten thousand epochs and then gradually converged to 0.02, meaning that the generator’s prediction accuracy improved quickly then converged. The generator’s fake score (blue line) in Figure 3f fluctuates around zero, which projects that the generator followed the development of the evaluator and had no model collapse during the training process.

### 2.3 cGAN performance evaluation

**Figure 4:**
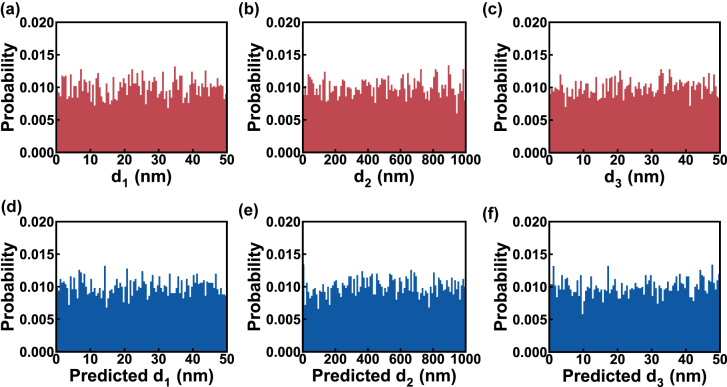
The distribution comparison between ground truth and prediction. The thickness histograms of (a–c) the testing set and (d–f) predicted by the generator.

After training, we used the testing set to evaluate the performance of the generator. The testing set contains 5000 data with the same uniform distribution as the training set (shown in Figure 4a–c). The trained generator takes the 5000 **Lab** and latent vectors **z** sampled from the standard normal distribution as inputs and predicts 5000 inverse designed thicknesses (denoted as **D**_p_). The distributions of the three thicknesses in **D**_p_ are plotted in Figure 4d–f. To quantify the distribution distance between predicted thickness and ground truth, the Jensen–Shannon distance (JSD) is introduced. The corresponding JSD computation process and reference values are provided in Supplementary Materials Section 4 (Figure S5 and Eqs. (S4)–(S8)). The measured JSDs for our case are 0.069 for *d*_1_, 0.067 for *d*_2_, and 0.066 for *d*_3_, reflecting that the predicted thickness and ground truth have great similarity in distribution. This proves that the trained generator is able to produce designs with restricted thickness distribution.

**Figure 5:**
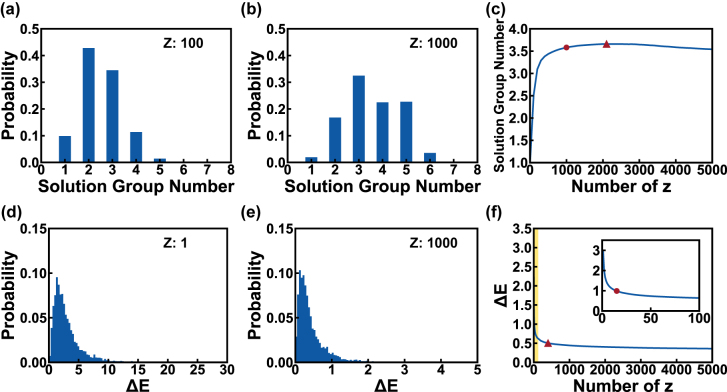
The tendencies of solution group number and Δ*E* as **z** sampling number varies. (a and b) The solution group number histograms when each **Lab** combines 100 (a) and 1000 **z** (b). (c) The curve of average testing solution group number against **z** number, the circle and triangle markers refer to the solution group numbers when each **Lab** assigned with 1000 and 2100 **z**, respectively. (d and e) The testing Δ*E* histograms when each **Lab** is assigned with 1 (d) and 1000 (e) **z**, respectively. (f) The curve of average testing Δ*E* against **z** number, in which the insert is the enlarge of yellow shading region, the circle and triangle markers refer to the Δ*E* while each **Lab** assigned with the **z** number of 15 and 400.

Next, we investigated the capability of the generator in achieving diverse solutions. As mentioned above, the latent vector **z** is introduced to generate multiple solutions for each **Lab**. Currently, only one **z** is sampled for each **Lab**, hence only one solution is predicted for each color. In order to obtain multiple solutions, we randomly sampled 1000 different **z** from the standard normal distribution for each **Lab** and classified the obtained 1000 solutions into different solution groups by the density-based spatial clustering of applications with noise (DBSCAN), an unsupervised machine learning clustering algorithm. Details of the classification process can be found in Section 5 of the Supplementary Materials. Figure 5a and b presents the distributions of solution group numbers when 100 and 1000 **z** were assigned to each **Lab**, respectively. The aggregation of solution group numbers moves from 1–4 to 2–5 as the **z** number grew from 100 to 1,000, showing a significant solution diversity improvement. An average of 3.58 solution groups can be identified for these 5000 **Lab** when each target color is assigned with 1000 different **z**, proving the capability of our network in achieving diverse solution groups. Figure 5c plots the solution group number as a function of different **z** numbers used for each color. It can be observed that the solution group number initially increases sharply with increasing **z** number and eventually saturates when the **z** number reaches over 1000 (highlighted by the red dot in Figure 5c). A maximum average solution number of 3.66 was obtained when 2100 **z** was used for each color (highlighted by the red triangle in Figure 5c). The following slight decrease is related to limitations in the clustering algorithm. As the **z** number increases, the different solution groups may slightly overlap with each other, which cannot be distinguished by DBSCAN.

Concurrently with the increasing number of solution groups, the color difference Δ*E* can also be significantly reduced. Figure 5d plots the Δ*E* distribution of 5000 solutions when one **z** is sampled for each **Lab**. The majority of the Δ*E* is located between 0 and 5, with an average Δ*E* value of 2.98. While 1000 **z** are sampled for each **Lab**, the lowest Δ*E* for each color almost falls in 0–1 (can be considered no color difference, as shown in Figure 5e), with an average value of 0.44. To the best of our knowledge, this is the lowest color difference reported for structural color inverse design. The dependence of the **z** number on the average lowest Δ*E* is presented in Figure 5f. An average lowest Δ*E* can reach below 1 when the **z** number is over 15 (highlighted by a red dot in Figure 5f inset) and below 0.5 when the **z** number is over 400 (highlighted by the red triangle in Figure 5f). To elucidate the high design accuracy achieved by our network, we compared the absolute *d*_2_ differences (Δ*d*_2_) between the solutions obtained from each color (using 1000 **z**) and its ground truth (global optimum). We discovered that 93.9% of the Δ*d*_2_ are within 5 nm, implying the majority of the ground truths have been successfully identified. Detailed distribution information of Δ*d*_2_ is provided in Figure S6.

All of these results suggest that both the quantity and quality of solutions can be improved using our network by increasing the number of **z**. It is worth mentioning that although multiple **z** sampling is required to achieve a high solution group number and low Δ*E*, the time required for such prediction is straightforward and largely effortless. For example, applying inverse design and solution optimization for 5000 colors with 1000 **z** sampling for each color will only consume 16.18 s in our computation environment, which is equivalent to 3.24 ms per color.

In order to verify the fabrication applications of multiple solution groups, we applied the generator to inversely design two colors generated by the F–P cavity. Table 1 lists the best solution for each obtained solution group. Both target colors have achieved more than two different solution groups. The ground truths (target designs) have been successfully identified as one of the solutions in both cases. In particular, five solution groups have been identified for color #2 with the *d*_2_ value spanning from 39 to 985 nm, proving the powerful searching ability of our network. These multiple solution groups allow the selection of suitable designs for the manufacturing requirement. Films that are too thin can be challenging to fabricate with high quality. For example, a design with a very thin Ag layer (e.g., 3 nm in design 3 for color #2) could increase the fabrication relative error and might need to be discarded despite its low Δ*E*. Instead, a different design (e.g. design 4) could be selected due to its relatively thicker films but still competitive Δ*E*. The sensitivity of the solution to fabrication errors is also an important factor to consider. We calculated the sensitivity here as the Δ*E* when *d*_2_ was added or subtracted by 1 nm (denoted as *δ*_1_ and *δ*_−1_, respectively). Higher *δ*_±1_ means that the solution is more sensitive to fabrication error and requires a more precise fabrication process. Although the ground truth of color #1 in Table 1 has been successfully identified in design 1, the sizeable *δ*_±1_ inflicts extreme requirements on the fabrication accuracy. On the other hand, design 3 with a slightly larger Δ*E* but the smallest *δ*_±1_ would be more suitable for the actual application. The capability of our network in identifying multiple solution groups for each color is clearly advantageous for providing viable designs for fabrication.

**Table 1:** The inverse design demonstrations of two colors. The target items are the ground truths. The designed items are from the solutions of the generator.

Target D (*d*_1_–*d*_2_–*d*_3_) nm	Target color (sRGB)	Designed D (*d*_1_–*d*_2_–*d*_3_) nm	Designed color (sRGB)	Comparison (target, design)	Δ*E*	(*δ*_1_, *δ*_−1_)
33–118–27	(−1.549)–0.534–0.426	34–118–26	(−1.481)–0.532–0.421	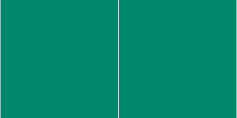	0.45	(2.03, 2.22)
		16–284–32	(−1.475)–0.536–0.420	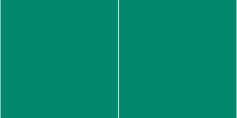	0.73	(1.03, 1.08)
		25–460–19	(−1.544)–0.528–0.417	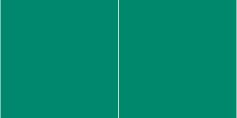	0.69	(0.60, 0.63)
3–550–41	0.302–0.274–0.453	21–39–18	0.193–0.268–0.470	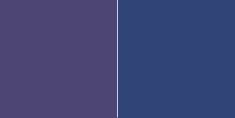	7.65	(0.19, 0.19)
		7–183–35	0.298–0.262–0.451	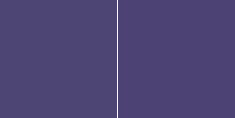	1.08	(0.44, 0.45)
		3–550–41	0.302–0.275–0.453	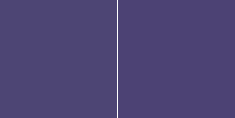	0.12	(0.30, 0.29)
		33–722–28	0.296–0.273–0.446	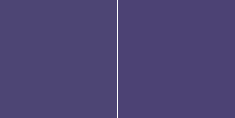	0.53	(0.42, 0.42)
		28–985–30	0.305–0.272–0.443	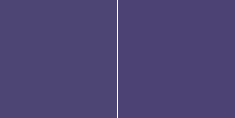	0.75	(0.27, 0.27)

We have proved from the discussion above that the proposed cGAN architecture can completely solve the “one-to-many” issue for this nanophotonics question. We argue that the vital component in this architecture is the evaluator, which enables the generator to learn the distribution of the dataset. Figure S8 presents the performance of the same networks with the evaluator disabled. The distributions of the three thicknesses designed for the testing set show no uniformity (Figure S8a–c). The measured JSDs between these designed thicknesses and the testing set are 0.21 for *d*_1_, 0.64 for *d*_2_ and 0.14 for *d*_3_, respectively. More importantly, with 1000 **z** sampled for each color, the network failed to identify multiple solution groups. Instead, the distribution of *d*_2_ is centralized between the range of 800–1000 nm, which leads to a relatively sizeable Δ*E* distribution (Figure S8d) with an average Δ*E* of 7.88 and a limited color coverage (Figure S8e). This proves that the evaluator plays a vital role in the solution quality and diversity of the generator. Finally, it is also worth pointing out that the balance between the generator and evaluator is also essential for the network performance (Figures S9–S11). A stronger discriminator will lead to a divergent generator, while a weaker discriminator will slow the training. Details are discussed in Section 4 of Supplementary Materials.

### 2.4 Experimental validation of designed sRGB color filters

**Figure 6:**
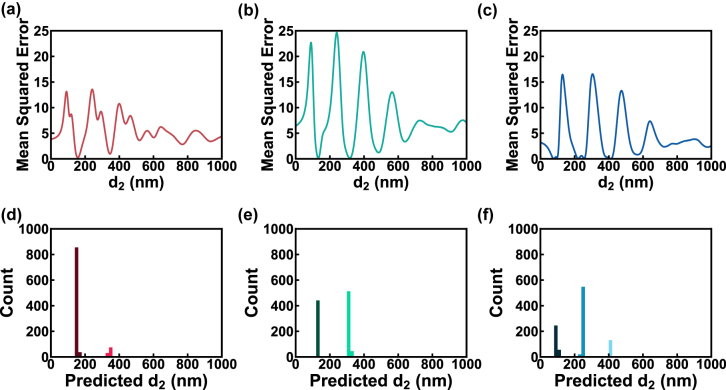
The analysis of sRGB color filter design results. (a–c) The MSE curves of the colors with sRGB values of (a) (0.5, 0, 0 red), (b) (0, 0.5, 0 green), and (c) (0, 0, 0.5 blue) when the SiO_2_ thickness (*d*_2_) was swept from 0 to 1000 nm and Ag thicknesses (*d*_1_ and *d*_3_) were fixed at 30 nm. The DBSCAN clustered predicted *d*_2_ histograms for the (d) red, (e) green and (f) blue colors. The dark-colored bar indicates the lower resonant order, and the light-colored bar means the higher order.

To further evaluate the network generalization and search performance of our network, we performed the inverse design of three color filters with sRGB values of red (0.5, 0, 0), green (0, 0.5, 0), and blue (0, 0, 0.5) using our trained generator with 1000 **z** sampling for each color. Note that these three colors are not presented in our dataset and hence have no ground truth. These examples could test the capability of our cCAN in searching multiply solutions for colors outside the dataset. To better visualize the searchability, we fixed the thicknesses of the top and bottom Ag layers to be 30 nm (this Ag restriction is removed in the cGAN inverse design) while sweeping the thickness of the SiO_2_ layer (*d*_2_) from 0 to 1000 nm to generate the loss (MSE) curves for each color (shown in Figure 6a–c). The loss between the target and swept colors varies as *d*_2_ changes. A small loss represents a better solution, and a dip towards 0 in the loss curve indicates one locally optimal solution group for the target. Therefore, the solution group number of each target color can be estimated from the number of curve dips towards 0. In this case, we could identify two solution groups for both the red and green colors, and three solution groups for the blue color. In addition, all the solution groups correspond to the different resonant orders for each color. The two solution groups at *ca.* 16 and 350 nm correspond to the 1st and 2nd order resonances for red (Figure 6a); the *ca.* 130 and 320 nm designs are the 1st and 2nd order resonances for green (Figure 6b); and the *ca.* 100, 250, and 410 nm designs are the first three resonant orders for blue (Figure 6c). The inverse design results of these three colors are demonstrated in Figure 6d–f. Here, we plot the distributions of the 1000 predicted *d*_2_ values for the three colors, respectively, in which *d*_2_ is uniformly divided into 100 groups in the range of 0–1000 nm and a bar indicates the solution number of each thickness group. Our network has successfully produced multiple solution groups for each color, matching the number and value of the solution groups identified from the loss curves. This suggests that our network can identify all possible solutions for different target colors. The relationships between the value of **z** and solution groups are discussed in Supplementary Materials (Figure S7).

**Figure 7:**
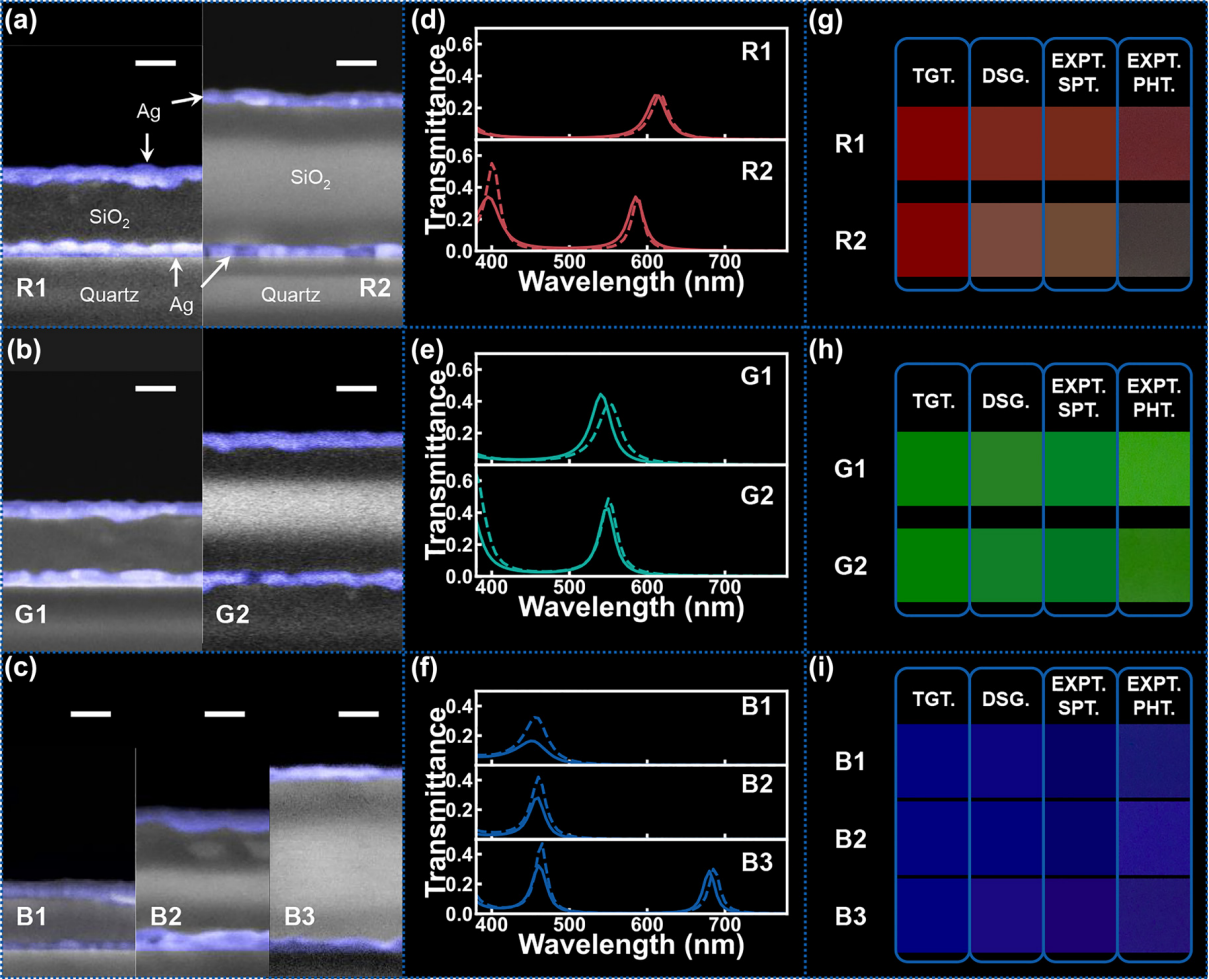
The experimental results of fabricated sRGB color filters. (a–c) The cross-sectional SEM images of the fabricated color filters with all the scale bars of 100 nm. (d–f) The measured spectra (solid line) and corresponding theoretical ones (dash line). (g–i) The color reconstructions, wherein TGT. is the target color; DSG. is the theoretical color of the designed color filter; EXPT. SPT. is the theoretical color of the measured spectrum and EXPT. PHT. is the photograph of the sample taken by the camera.

We will now experimentally validate our multiple solution groups by fabricating the red, green, and blue color filters previously designed by our cGAN. The parameters of the best solution in each solution group for each color are listed in Table 2. All the designed color filters were fabricated on quartz substrates. Details of the fabrication process are available in the method section. Figure 7a–c displays the cross-sectional SEM of all fabricated samples. A three-layered structure containing Ag (blue shading) top and bottom layers and SiO_2_ dielectric is clearly visible for all samples. It is also evident that the dimensions (especially the thickness of the SiO_2_ layer) are significantly different for different designs of the same target color. The measured transmission spectra for all samples are compared with their theoretical (calculated from designed parameters) counterparts and shown in Figure 7d–f. All measured spectra match well with the theoretical ones in terms of both transmission peak wavelength and intensity. The discrepancies in peak intensity at shorter wavelengths may be attributed to the surface oxidization of the Ag films [60, 61]. Figure 7g–i displays the resultant colors for each design. For each target color (TGT.), we compare the theoretical colors from different designs (DSG.) with the colors converted from corresponding experimental spectra (EXPT. SPT.) and the photographs of the samples taken by the camera under a sunlight-like source (EXPT. PHT.). The discrepancy in the R2 photograph may result from the light source intensity or the camera parameters (e.g., white balance, contrast, exposure time, etc.).

**Table 2:** The targets and parameters of designed sRGB color filters.

Sample name	Target color (sRGB)	Designed D (*d*_1_–*d*_2_–*d*_3_) nm	Designed lab	Designed sRGB	Δ*E*	(*δ*_1_, *δ*_−1_)
R1	0.5–0–0	37–158–38	26.2–34.7–26.7	0.449–0.134–0.090	5.20	(1.30, 1.38)
R2	0.5–0–0	37–346–32	29.9–17.4–14.4	0.399–0.233–0.192	13.81	(2.09, 2.22)
G1	0–0.5–0	33–134–36	46.7–(−45.2)–41.4	0.141–0.499–0.130	2.77	(1.74, 1.64)
G2	0–0.5–0	28–318–31	45.9–(−46.7)–37.5	0.076–0.494–0.158	3.92	(0.86, 0.83)
B1	0–0–0.5	30–99–49	13.9–47.3–(−65.6)	(−0.011)–0.012–0.517	0.79	(1.67, 1.44)
B2	0–0–0.5	40–256–31	12.4–49.0–(−65.8)	(−0.014)–(−0.013)–0.503	0.57	(1.02, 0.94)
B3	0–0–0.5	36–414–30	13.8–43.9–(−59.6)	0.080–0.024–0.480	1.48	(0.79, 0.74)

## 3 Conclusions

In conclusion, a variation of cGAN has been successfully implemented to resolve the typical one-to-many problem in nanophotonics using the inverse design of the F–P-cavity-based color filter as a demonstration. We suggest that the key to avoiding solution group degeneracy is generating solutions with the same parameter distribution of a dataset covering most solution spaces. Equipped with an evaluator that enables learning the dataset distribution, the cGAN developed in this work is able to identify an average of 3.58 solution groups for each color. Simultaneous with the improved solution diversity, the quality of the solutions is also significantly improved. An average Δ*E* of 0.44 has been achieved when sampling 1000 **z** for each color. The high design accuracy could be attributed to identifying 93.9% ground truths of the testing set. Finding multiple solution groups can also benefit design manufacturing to allow more viable designs for fabrication. The capability of our cGAN has also been verified experimentally by inverse designing the sRGB color filters. The measured spectra and photographs of fabricated two red, two green, and three blue color filters present excellent agreement with the designs. It should be acknowledged that the presented work is limited by its dataset range as well as its simple three-layered structure. However, it is worth highlighting that the three-layer F–P cavity structure only serves as an example to demonstrate the capability of cGAN and the novelties of this work remain valid. We envisage this cGAN-based design methodology to be applied to other nanophotonic structures or physical science domains where the identification of multi-solution across a vast parameter space is required.

## 4 Methods

### 4.1 Fabrication

Before fabricating the device, a series of experiments were conducted to identify the deposition rate for both Ag and SiO_2_ using both ellipsometry (Woollam M-2000DI) and atomic force microscopy (Park System X7) for calibration. The same recipes were then used to fabricate our designed structural colors to ensure the best experimental reproduction of the designed structures. During the fabrication of structural colors, the bottom Ag thin film was firstly deposited by the electron-beam evaporation system (Leybold LAB700) with a growth rate of 0.5 Å/s. Without breaking the vacuum, a 1 nm thin Ti film was formed by electron-beam evaporation with a growth rate of 0.1 Å/s on top of the Ag film to improve the adhesion between Ag and the following SiO_2_. The SiO_2_ layer was subsequently formed on the Ti layer in a reactive sputtering system (Leybold Helios Pro XL) using Si target and O_2_ plasma. The growth rate of SiO_2_ is 0.52 nm/s. After completing the SiO_2_ deposition, the top Ag film was formed with the same process condition as the bottom Ag film and then a 1 nm Ti film on top.

### 4.2 Characterization

The refractive index of SiO_2_ used in the calculation is measured by an ellipsometer (Woollam M-2000DI). The transmission spectra under normal incidence were measured by a spectrometer (Bentham PVE300) with Air as reference. The photographs were taken using a digital camera (Canon EOS 500D) with a shutter speed of 1/20 s, an aperture of f/5, and the default white balance. In order to produce the source with the correct color temperature, an Apple iPad Pro (10.5 inch, 2017 year version) was used as a backlight source, with both true tone and night shift switched off and brightness set as maximum. The original photograph is shown in Figure S13. The cross-sectional morphologies of fabricated multiple thin-film structures were characterized using a field-emission scanning electron microscope (JEOL 7500 FEGSEM) at an acceleration voltage of 2 kV and a working distance of 4 mm.

### 4.3 Calculation

All the calculations and neural network training were performed on a Windows PC with the hardware: CPU: Intel Core i9-9900K; RAM: 48 GB; GPU: NVIDIA RTX 2070. The involved software packages include Python 3.8, PyTorch 1.9.0, NumPy, Scikit-Learn and Colour-Science. The refractive index of Ag used in the calculation is Ag (Palik 0–2 μm) [62].

## Supplementary Material

Supplementary Material
