# Supplementary material for: Inverse design of structural color: finding multiple solutions via conditional generative adversarial networks
Source: Nanophotonics. 2022 May 16;11(13):3057–69. doi: 10.1515/nanoph-2022-0095 (PMC11501759; doi:10.1515/nanoph-2022-0095)
Supplement: Supplementary file 1 — Supplementary Material [file j_nanoph-2022-0095_suppl.docx]

# Supplementary Materials of Inverse Design of Structural Color: Finding Multiple Solutions *via* Conditional Generative Adversarial Networks

Peng Dai^1^, Kai Sun^1^, Xingzhao Yan^1^, Otto L. Muskens^1^, C.H. (Kees) de Groot^1^, Xupeng Zhu^3^, Yueqiang Hu^2^, Huigao Duan^2^, Ruomeng Huang^1^*

^1^Faculty of Engineering and Physical Sciences, University of Southampton, Southampton, United Kingdom, SO17 1BJ

^2^National Engineering Research Center for High Efficiency Grinding, College of Mechanical and Vehicle Engineering, Hunan University, Changsha, China, 410082

^3^School of Physics Science and Technology, Lingnan Normal University, Zhanjiang, China, 524048

[*r.huang@soton.ac.uk](mailto:*r.huang@soton.ac.uk)

These authors contributed equally: Peng Dai, Kai Sun

## Neural Network Architecture

Figure S1 illustrates the neural network architecture details used in this paper. Figure S1a presents the linear block, which includes a sequence of one fully connected layer, one 1D batch normalization layer, and finally following a LeakyReLU activation function. Figure S1b shows the detail of Lab regressor. It has seven layers of the linear block with the hidden neuron number of 256 and the input and output dimension of 3. The network of the generator is shown in Figure S1c. The generator has two linear blocks as the upraising layers to increase the dimensions of the latent vector and color vector from 2 and 3 to 128. The upraised vectors are then concatenated with the dimension of 256. The combined vector is finally fed to the thickness regressor, which has nine layers of the linear block, one fully connected layer, and a sigmoid activation function to scale the output value in the range of 0-1. Figure S1d is the architecture schematic of the distribution evaluator. This network employs five layers of the linear block, which precedes a dropout layer with a probability of 0.9 and a fully connected layer at the end to output the distribution score. All the fully connected layers in linear blocks and networks are applied with spectral normalization to improve the performance and stability.

Figure S1. a) The schematic of the linear block. b) The schematic of Lab regressor. c) The schematic of the generator. d) The schematic of the evaluator.

## Dataset Generation

Figure S2 illustrates the obtained color coverages on CIE 1931-xy chromatic diagram, while d_1_, d_2_ and d_3_ were sampled from a uniform distribution with the range of 0-50 nm, 0-1000 nm, and 0-50 nm. Then, we measured the Pearson correlation efficient heatmap of parameters (**D**) and color (**HSV**), which is plotted in Figure S3a. From it, we could observe that d_1_ and d_3_ show a strong negative correlation with value, positive correlation with saturation, and weak positive correlation with hue, which indicate that the increase of d_1_ and d_3_ will reduce the brightness of color but benefits the saturation, and slightly increase hue (blue shift for spectrum). This is reasonable since the tri-layer structure consists of a Fabry-Perot cavity. The thicker Ag films will gain higher reflectance, resulting in narrower transmission peaks and less energy. Therefore, a darker but pure color is produced (Figure S3b). Unlike the previous two parameters, d_2_ has few affections to value but plays more critical roles in saturation and hue. There are two possible reasons for this: 1) SiO_2_ worked as a spacer in the F-P cavity, so its thickness (d_2_) determined the distance between two mirrors (Ag films), the number of transmission peaks in the visible range will rise as the increase of d_2_, and this means that more colors appear simultaneously and cross-talked with each other, so the saturation of color experiences a degradation. 2) The increase of d_2_ will result in a redshift (decrease of hue) of the transmission spectrum in the small thickness value region, for example, tens to around 200 nm. However, if we switch the view of point to span 0-1000 nm because the resonant wavelength of light is the integer times of half effective wavelength in SiO_2_, so short wavelength color (e.g., violet, blue, and cyan etc.) will appear more frequently. This can lead to the appearance of color showing more blue-like and the growth of hue. To verify our analysis, we splatted the obtained colors in the dataset into three parts, red, green, and blue, by the k-nearest neighbor (KNN) search and plotted the corresponding d_2_ histograms in Figure S3(f-h). From these figures, we can observe that almost d_2_ larger than 500 nm is classified as blue, which can give rise to the value of the hue.

Figure S2. The CIE 1931-xy chromaticity diagrams. a) The CIE 1931-xy chromaticity diagram of the training and validation sets. b) The CIE 1931-xy chromaticity diagram of the testing set.

Figure S3. The relationship figures between thickness and color. a) The heatmap between **HSV** and **D**. b) The spectra while SiO_2_ is fixed as 150 nm with different Ag film thicknesses. c-e) The splatted color coverages in CIE 1931-xy chromaticity diagrams for red, green, and blue. f-h) The splatted d_2_ distributions for red, green, and blue.

## Neural Network Training

Table S1 shows the training parameters. The Lab regressor was trained with an Adam optimizer for 10,000 epochs. The batch size was tuned to the largest (40,000) to boost the training speed. The learning rate is set as $5\times{10}^{-3}$ in the first 5,000 epoch, then linearly decayed to 0, making the network smoothly converge. Therefore, the trained Lab regressor has a state-of-the-art color prediction performance with an average testing ΔE of 0.19. The total regressor training wall-clock time is 302.8 s (5 min).

Table S1. The training hyperparameters of Lab regressor.

Figure S4a presents the training and validation loss curves. The training and validation losses decreased sharply in the first 1,000 epochs, from then on, fluctuating around $5\times{10}^{-3}$ util 5,000^th^ epoch, which means the performance is saturated in this learning rate. Finally, the losses gradually converge to $1.5\times{10}^{-4}$ as the learning rate decline. Figure S4b shows testing ΔE distribution. As we can see, almost the testing ΔE is less than 0.5, inferring the excellent prediction performance of the Lab regressor.

Figure S4. The training results of Lab regressor. a) The training and validation loss curves of the Lab regressor. b) The histogram of Lab regressor testing ΔE.

Table S2 shows the training hyperparameters of cGAN. The generator and evaluator were trained for 100,000 epochs with the update step ratio of 1:1. To prevent the evaluator trained too powerful making the generator unable to follow, the learning rate of the evaluator is set as 1/5 of the generator. The batch size is set to the largest, 40,000, to accelerate the training rate. The parameters of the Adam optimizer are customized ($\beta_{1}=0.5$ and $\beta_{2}=0.999$) to adopt the GAN task. The random seed is fixed as 42 to guarantee repeatability. The GAN training wall-clock time is 3.7 h.

Table S2. The training hyperparameters of GAN.

The loss functions used in the training process are listed in Equation S1-S3, where $L_{E}$ and $L_{G}$ are the losses of the evaluator and the generator; $S_{Er}$ and $S_{Ef}$ are the real and fake scores predicted by the evaluator for evaluator update; $S_{Gf}$ is the fake score predicted by the evaluator for generator update; $\alpha$ is a dynamic weight to balance the distribution and regression abilities of the generator; $\text{Eval}$ refers to the evaluator of the discriminator; $N(0,1)$ is the normal distribution with the average value of 0 and standard deviation of 1; $\text{Gen}$ is the generator; $\text{LR}$ presents the Lab regressor; $\boldsymbol{D}_{r}$ is the thickness vectors of ground truth; $\boldsymbol{D}_{Ef}$ and $\boldsymbol{D}_{Gf}$ is the thickness vectors predicted by the generator for updating the evaluator and generator, respectively; $\boldsymbol{z}_{E}$ and $\boldsymbol{z}_{G}$ are the latent vectors sampled from the normal distribution; $\boldsymbol{S}_{Er}$ is the real score vector; $\boldsymbol{S}_{Ef}$ and $\boldsymbol{S}_{Gf}$ are the fake score vectors for updating the evaluator and generator; $\boldsymbol{S}_{Ef}$ and $\boldsymbol{S}_{Gf}$ are the generator’s fake score vectors for updating the evaluator and generator; $\boldsymbol{Lab}_{r}$ is the Lab vectors of ground truth; $\boldsymbol{Lab}_{f}$ is the Lab vectors predicted by the Lab regressor.

| $\left\{ \begin{aligned} &L_{E}=S_{Er}+S_{Ef} \\ &L_{G}=S_{Gf}+\alpha\text{MSE}\left( \boldsymbol{Lab}_{r},\boldsymbol{Lab}_{f} \right) \end{aligned} \right.$ | (S1) |
| --- | --- |
| $\left\{ \begin{aligned} &S_{Er}=\frac{1}{n}\sum_{i} \text{ReLU}\left( 1-S_{Eri} \right) \\ &S_{Ef}=\frac{1}{n}\sum_{i} \text{ReLU}\left( 1+S_{Efi} \right) \\ &S_{Gf}=-\frac{1}{n}\sum_{i} S_{Gfi} \\ &\alpha=\text{Min}(1,\frac{epoch}{20000}) \end{aligned} \right.$ | (S2) |
| $\left\{ \begin{aligned} &\boldsymbol{S}_{Er}=\text{Eval}\left( \boldsymbol{D}_{r} \right) \\ &\boldsymbol{z}_{E}\sim N\left( 0, 1 \right) \\ &\boldsymbol{D}_{Ef}=\text{Gen}\left( \boldsymbol{Lab}_{r},\boldsymbol{z}_{E} \right) \\ &\boldsymbol{S}_{Ef}=\text{Eval}\left( \boldsymbol{D}_{Ef} \right) \\ &\boldsymbol{z}_{G}\sim N\left( 0, 1 \right) \\ &\boldsymbol{D}_{Gf}=\text{Gen}\left( \boldsymbol{Lab}_{r},\boldsymbol{z}_{G} \right) \\ &\boldsymbol{S}_{Gf}=\text{Eval}(\boldsymbol{D}_{Gf}) \\ &\boldsymbol{Lab}_{f}=\text{LR}\left( \boldsymbol{D}_{Gf} \right) \end{aligned} \right.$ | (S3) |

## Neural Network Evaluation

The calculation process of Jessen-Shannon distance in this paper is following:

If there are two probability distributions, $P(x)$ and $Q(x)$, the JS distance is given by

| $JSD(P\vert\vert Q)=\sqrt{\frac{1}{2}D\left( P\vert\vert M \right)+\frac{1}{2}D\left( Q\vert\vert M \right)}$ | (S4) |
| --- | --- |
| $\left\{ \begin{aligned} &M\left( x \right)=\frac{1}{2}\left( P\left( x \right)+Q\left( x \right) \right) \\ &D\left( P\vert\vert M \right)=\sum_{x} P\left( x \right)\log\frac{P\left( x \right)}{M\left( x \right)} \\ &D\left( Q\vert\vert M \right)=\sum_{x} Q\left( x \right)\log\frac{Q\left( x \right)}{M\left( x \right)} \end{aligned} \right.$ | (S5) |

Regarding to the calculation detail of our case, we uniformly divided the thicknesses, d_1_, d_2_ and d_3_ into 100 groups in the ranges of 0-50 nm, 0-1,000 nm, and 0-50 nm, respectively. The number in each group was then counted and divided by 5,000 (the total number of the testing set). We, hence, achieved the probability density in each thickness group. We denoted the predicted thickness probability distribution as $Q(q_{i},q_{2},\ldots,q_{100})$ and the true thickness probability distribution as $P(p_{1},p_{2},\ldots,p_{100})$, where $q_{i}$ and $p_{i}$ are the probability density of each group for predicted thickness and true thickness, respectively. The JSD is then calculated as previous Equations:

| $\left\{ \begin{aligned} &m_{i}=\frac{1}{2}\left( p_{i}+q_{i} \right) \\ &M=\left( m_{1},m_{2},\ldots,m_{100} \right) \end{aligned} \right.$ | (S6) |
| --- | --- |
| $\left\{ \begin{aligned} &D\left( P\vert\vert M \right)=\sum_{i=1}^{100} p_{i}\log\frac{p_{i}}{m_{i}} \\ &D\left( Q\vert\vert M \right)=\sum_{i=1}^{100} q_{i}\log\frac{q_{i}}{m_{i}} \end{aligned} \right.$ | (S7) |
| $JSD=\sqrt{\frac{1}{2}D\left( P\vert\vert M \right)+\frac{1}{2}D\left( Q\vert\vert M \right)}$ | (S8) |

The obtained JSD between predicted thickness and true thickness are 0.069, 0.067, and 0.066 for d_1_, d_2_ and d_3_, respectively, projecting the great distribution similarity.

There are some examples of the JSD between different distributions (as shown in Figure S5). The JSD for Figure S5a to S5c are 0, 0.17, and 0.33. The JSD for Figure S5d to S5f are 0, 0.29, and 0.42. Figure S5a to S5c are the comparisons between two normal distributions, in which the blue lines are the curves of the normal distribution with the average value ($\mu)$ of 0 and the standard deviation ($\sigma)$ value of 1, the red lines are the average value of 0, 0.5, and 1, and the standard deviation of 1, respectively. Figure S5d to S5f are the comparisons between two uniform distributions, in which the blue lines are the uniform distributions with the average value of 0, and the probability density of 0.25, the red lines are the average value of 0, 0.5, and 1, and the probability density of 0.25, respectively.

Figure S5. The JSD comparison examples between normal distributions (a-c) and uniform distributions (d-f).

Figure S6 presents the minimum dielectric thickness differences (Δd_2_) between the predictions (${\hat{\boldsymbol{d}}}_{2}$) and the corresponding ground truth ($d_{2}$) in the testing set, in which **z** is sampled 1,000 times for each **Lab**. Wherein, 83.2% Δd_2_ is less than 1 nm (on the left of the red line), 93.9% less than 5 nm (on the left of the yellow line), and 96.7% less than 10 nm (on the left of the green line). These data indicate that the trained cGAN found almost all global optima during the inverse design in the testing set.

| $\left\{ \begin{aligned} &{\boldsymbol{\Delta}\boldsymbol{d}}_{2}=\left\vert{\hat{\boldsymbol{d}}}_{2}-d_{2} \right\vert\\ &\Delta d_{2}=\text{min}\left( \boldsymbol{\Delta}\boldsymbol{d}_{2} \right) \end{aligned} \right.$ | (S9) |
| --- | --- |

Figures S6. The Δd_2_ histograms between predicted d_2_ and ground truths in the testing set.

The relationship between the solution and the value of **z** is explored. We plotted the ΔE distributions (Figure S7g-S7i) of the designed sRGB color filters in the main text and the scatter figures of **z** (Figure S7j-S7l)). The colored background in scatter figures illustrates the probability density of **z** sampling, the redder zone indicates the higher sampling probability, and white refers to the lower. We labeled the bars in Figure S7g-S7i and scatters in Figure S7j-S7l with different color brightness to signify the different solutions. It can be noticed that the generator divides the space of **z** according to the input **Lab**. The pleasing designs (lower ΔE) are assigned to the large probability density regions. The dreadful ones (large ΔE) are distributed in the low probability density areas, indicating that the better solutions are more likely to be found. This is reasonable since the generator has to decrease the color difference in the training process, improving the possibility of reasonable solutions and decreasing the bad ones. However, the probability of bad ones will not be zero because the distribution part of the loss function forces its output across the entire design space to keep the solution diversity.

Figure S7. The relationship between ΔE and the value of **z**. a-c) The MSE curves between the swept colors, while Ag films fixed as 30 nm and SiO_2_ varied from 0 to 1000 nm, and the colors with sRGB values of red (0.5, 0, 0) (a), green (0, 0.5, 0) (b) and blue (0, 0, 0.5) (c). d-f) The DBSCAN clustered predicted d_2_ histograms, wherein the dark color indicates the lower resonant order and the light color means a higher one. g-i) The ΔE distributions of obtained solutions. j-l) The scatter figures of **z**, wherein the colored background indicates the sampling probability density, and the dots are the corresponding **z** of obtained solutions.

Figure S8 presents the testing results while the evaluator is removed from the discriminator. Figure S8a-S8c show the testing predicted thicknesses distributions. The measured JSD for the d_1_, d_2_, and d_3_ with the testing set distribution are 0.21, 0.64, and 0.14, respectively. From these data, we can notice that predicted thicknesses, especially d_2_, concentrated on a small region and have a significant distribution difference with ground truth, leading to a relatively sizeable ΔE distribution (Figure S8d) with an average ΔE of 7.88 a small color coverage (Figure S8e). This indicates that the evaluator is crucial to force the solutions of the generator spread all the solution space and play a vital role in the solution quality and diversity.

Figure S8. The testing results of cGAN when evaluator disabled. a-c) The testing predicted thickness distribution while the evaluator is disabled. d) The corresponding testing ΔE distribution. e) The corresponding predicted color distribution on CIE 1931-xy chromaticity diagram.

Figure S9 illustrates the training loss curves while the evaluator has more two layers of linear blocks than the balance network. Figure S9a-S9c shows that the discriminator and generator losses oscillated and diverged, reflecting a mode collapse during training. The irregular predicted thickness distributions (Figure S9d-S9f) also confirmed the results.

Figure S9. The training and testing results that the evaluator has more two layers of linear block. a) The loss curves of GAN while evaluator has more two hidden layers than the network used in the main text of this paper. b) The curves of the evaluator detail loss components, which are real (red) and fake (blue) scores. c) The curves of the generator’s detail loss components, MSE between predicted **Lab** and ground truth (red) and fake score (blue). d-f) The generator predicted thickness distributions.

Figure S10 gives another imbalance example the evaluator has less two layers of linear blocks. The training loss curves in Figure S10a-S10c show a similar trend to the balance network in the main text of this paper, but the predicted thickness distributions (Figure S10d-S10f) present less uniformity than the ground truth. This reflects that the generator is not fully trained, indicating that a too-small evaluator network size may reduce the training rate of the generator. Therefore, more time and computation resources are required to complete the GAN training.

Figure S10. The training and testing results that the evaluator has less two layers of linear block. a) The loss curves of GAN while evaluator has less two hidden layers than the network used in the main text of this paper. b) The curves of the evaluator detail loss components, which are real (red) and fake (blue) scores. c) The curves of the generator’s detail loss components, MSE between predicted **Lab** and ground truth (red), and fake score. d-f) The generator predicted thickness distributions.

Figure S11 gives solution diversity and quality of two imbalance examples. Figure S11a presents the solution number distribution of two examples. Both can only predict one solution no matter how **z** varies. Figure S11b is the testing ΔE distribution when the evaluator has more two layers of linear blocks, wherein almost the testing ΔE are mainly less than 40 with an average of 9.68 while sampling 1,000 **z** for each **Lab**. Figure S11c shows the testing ΔE distribution when the evaluator has less two layers of linear blocks, almost testing ΔE are mainly less than 20 with the average of 5.26 while sampling 1,000 **z** for each **Lab**. The two imbalance examples prove that the network size of the generator and evaluator is a crucial fact making the generator converge at a great point.

Figure S11. The multiple solution testing results which the evaluator and generator are imbalanced. a) The solution group number histograms when the evaluator has more or less two layers of linear blocks, and 1,000 different **z** is sampled for each color. The ΔE distributions when the evaluator has less b) and more c) two layers of linear blocks, in which the **z** sampling times is 1,000.

## DBSCAN

Table S4 presents the hyperparameters of density-based spatial clustering application with noise (DBSCAN). DBSCAN was designed to cluster high-dimensional data with discretionary shapes and noise. The fundamental concept of DBSCAN is that for each clustering object, the neighborhood with a radius (ε) must include a minimum number of objects ($N_{min}$), indicating that the number of neighborhoods has to surpass some threshold. The ε-neighborhood of a point $p$ is defined as,

$$N_{\epsilon}=\{dist(p,q)<\epsilon|q\in D\}$$

Where, $D$ is the database of objects. If the ε-neighborhood contains at least a minimal number of points ($N_{min}$), and then the point $p$ is considered a core point. The core point is defined as: $N_{\epsilon}\left( p \right)>N_{min}$, where $\epsilon$ and $N_{min}$ are user-defined hyperparameters. When this condition has not met the threshold, the point is considered a non-core point, which will be waived in the clustering process.^1^

Table S4. The hyperparameter used in DBSCAN

Figure S12 shows some examples of the clustering algorithm. We randomly selected three colors from the testing set and put them into the trained generator with 1,000 **z** to get the solutions (shown in Figure S12a-S12c). DBSCAN then clusters the obtained d_2_ predictions using the hyperparameters in Table S4. The bars with different colors in Figure S12d-S12f mean the different solution groups for each color. The clustering results show that the algorithm successfully classified the different solution groups.

Figure S12. Some examples of DBSCAN. a-c) The predicted d_2_ histograms of three colors randomly selected from the testing set. d-f) The DBSCAN clustered d_2_ histograms.

## Experimental results

Figure S13 presents the original photograph taken by the camera.

Figure S13. The photograph of samples taken by the camera.

## Reference:

(1) Khan, K.; Rehman, S. U.; Aziz, K.; Fong, S.; Sarasvady, S. DBSCAN: Past, Present and Future; IEEE, 2014; pp 232–238.
